# Catarrhal Phthisis in a Llama, with a Fibro-Calcareous Tumor in the Perineal Region

**Published:** 1882-10

**Authors:** Wm. A. Conklin


### IV.—CATARRHAL PHTHISIS IN A LLAMA WITH A FIBRO-CALCAREOUS TUMOR IN THE PERINEAL REGION.

REPORTED FROM THE CENTRAL PARK ZOOLOGICAL GARDEN BY WM. A. CONKLIN, D.V.S.

The first symptoms occurred July 13th, 1882, when it was noticed that the animal in question refused food. It also maintained a sitting posture, and could not be induced to rise.

July 14th it appeared a little better, and tried to eat, but could not be induced to walk around or take any exercise.

July 15th an anæma of soap and water was used to move the bowels.

When the attendant was administering the enema, he noticed for the first time a large swelling alongside the sheath of the penis.

Upon physical examination of the tumor, it was found to be hard and quite freely movable. Manipulation of the neoplasm did not cause pain; in fact, the new growth caused no disturbance whatever.

On the morning of July 16th the animal suddenly died.

*Necropsy.*—The Llama was in fair condition.

*Thoracic Cavity.*—The heart appeared to be normal in every respect. The lungs, however, showed marked evidence of a phthisical process in the superior lobes. The inferior lobes were deeply congested and œdematous. The tumor, which lay alongside the sheath, had no connection with the penis, but seemed to lie between it and the muscles of the thigh.

The growth was nearly circular in outline and quite hard, as if bony. It was ten centimetres, or four inches, in diameter. The minute structure of the growth was fibrous, with a shell of calcareous matter. A fibrous tumor, with a crust of calcareous matter. In all probability this new growth had nothing to do with the cause of death.

The cause of death, I think, might more accurately be ascribed to the phthisical process, associated with a sudden congestion and œdema of the lungs, causing a paralysis of the heart.

NEW YORK CITY, July, 1882.

